# Hepatic immune microenvironment dynamics in severe obesity and post-bariatric surgery: insights from immunohistochemical analysis

**DOI:** 10.3389/fimmu.2026.1816648

**Published:** 2026-09-03

**Authors:** Alina-Iuliana Onoiu, Sergio González-Serrano, Vicente Cambra-Cortés, Andrea Jiménez-Franco, Carmen Guilarte, Karla Beatriz Peña, Francesc Riu, Jordi Camps, Jorge Joven, David Parada

**Affiliations:** 1 Unitat de Recerca Biomèdica, Hospital Universitari Sant Joan, Reus, Spain; 2 Department of Medicine and Surgery, Faculty of Medicine, Universitat Rovira i Virgili, Reus, Spain; 3 Institut d’Investigació Sanitària Pere Virgili, Tarragona, Spain; 4 Department of Pathology, Hospital Universitari Sant Joan, Reus, Spain; 5 The Campus of International Excellence Southern Catalonia, Tarragona, Spain

**Keywords:** digital pathology, liver inflammation, MASH resolution, neutrophils, QuPath

## Abstract

Severe obesity is linked to metabolic dysfunction-associated steatotic liver disease (MASLD), which can progress to metabolic dysfunction-associated steatohepatitis (MASH) and advanced fibrosis. Currently, there are no accurate tissue biomarkers for measuring immune-mediated liver injury or remission post-bariatric surgery. This study utilized digital pathology and immunohistochemistry to analyze liver biopsies from three patient groups: those with severe obesity and liver damage before bariatric surgery, their paired re-do biopsies post-surgery, and patients with severe obesity and without evaluable liver injury per NASH CRN criteria. Quantitative metrics assessed immune cell proportions and staining areas from whole-slide images. Following bariatric surgery, patients experienced a significant reduction in body mass index (BMI) and improvements in liver histology, although lobular inflammation persisted at a median follow-up of six years. Analysis of the innate immune response showed a notable increase in CD15^+^ neutrophils postoperatively, along with a progressive accumulation of CD56^+^ natural killer cells across healthy and pre- and post-surgical cohorts. Additionally, long-term post-surgical samples exhibited higher densities of CD68^+^ and CD163^+^ macrophages. Within the adaptive immune compartment, the number of CD4^+^ T cells significantly increased after surgery, while the abundance of CD8^+^ T cells remained stable across all groups. Overall, the livers of postsurgical patients displayed the highest burden of immune cells and a restructured hierarchy of immune populations. This indicates the presence of a unique immune microenvironment associated with surgery rather than a return to a “healthy liver” profile. Despite metabolic and histological improvements post-surgery, persistent immune activation suggests a unique immunological state where immune cells may play dual roles in both liver damage and repair, emphasizing the need for comprehensive immune profiling and targeted therapeutic strategies.

## 1 Introduction

Obesity is one of the most widespread health issues globally, affecting over 890 million adults in 2022. It poses a significant public health challenge in the 21st century. Since 1990, the prevalence of obesity has nearly tripled, with severe obesity (defined as a BMI of 40 kg/m² or higher) showing particularly concerning increases (1, 2). This epidemic has serious implications for healthcare systems around the world, as obesity is linked to numerous comorbidities, including type 2 diabetes, cardiovascular disease, and liver disease (3).

When conservative treatments do not result in sustained weight loss for individuals with severe obesity, surgical intervention becomes necessary (4, 5). Sleeve gastrectomy has emerged as one of the most commonly performed bariatric procedures worldwide (6). It has proven to be highly effective not only for achieving substantial weight loss but also for improving obesity-related conditions, including hepatic steatosis and inflammation (7–10). However, some patients may require a second re-do bariatric intervention due to insufficient initial weight loss or significant weight regain over time (11).

Metabolic dysfunction–associated steatotic liver disease (MASLD) affects up to 75% of severely obese individuals, representing one of the major comorbidities in this population (12). MASLD encompasses a spectrum of liver pathology ranging from simple steatosis to metabolic dysfunction-associated steatohepatitis (MASH), which is characterized by hepatocyte ballooning, lobular inflammation, and varying degrees of fibrosis. Early identification and treatment of MASLD and MASH are crucial to prevent progression to irreversible conditions such as cirrhosis, hepatocellular carcinoma, and end-stage liver disease that significantly increase liver-related mortality.

The immune system plays a pivotal role in both the pathogenesis and progression of MASH, as well as in its potential tissue repair following therapeutic interventions (13, 14). The hepatic immune microenvironment in MASH is characterized by a complex interplay between resident immune cells, including Kupffer cells and dendritic cells, and infiltrating immune populations such as monocyte-derived macrophages (MDM), T cells, and neutrophils (15). Altered immune cell infiltration in liver tissue is associated with more severe histological damage and advanced disease stages (16). During disease progression, pro-inflammatory macrophages (M1 phenotype) predominate, releasing inflammatory cytokines including Tumor Necrosis Factor-α (TNF-α), Interleukin-1β (IL-1β), and IL-6, which perpetuate hepatic inflammation and promote fibrogenesis. Conversely, anti-inflammatory M2 macrophages are crucial for tissue repair and inflammation resolution (17, 18). The dynamic balance between these immune cell populations and their functional states determines disease course, making immune modulation a key mechanism underlying treatment success (19, 20).

Given the importance of the immune system in disease progression and remission, traditional pathological evaluation methods, which rely on manual counting of inflammatory foci in 20x fields to assess lobular inflammation scores, may not fully capture the complexity of the hepatic immune microenvironment (21). Digital pathology has revolutionized liver histology analysis by enabling precise quantification of immune cell populations and their spatial distribution (22–24).

In this study, we employed digital pathology combined with immunohistochemical techniques to comprehensively characterize the hepatic immune microenvironment in severe obesity across different clinical contexts. We analyzed liver tissue from two distinct patient cohorts: severely obese patients with histologically evident liver alterations and their corresponding re-do liver biopsies following bariatric intervention, and severely obese patients without evaluable liver alterations with Nonalcoholic Steatohepatitis Clinical Research Network (NASH CRN) Score of 0. This comparative approach allows for detailed examination of immune cell dynamics across the spectrum of liver health in severe obesity, from normal hepatic architecture through disease progression to post-surgical resolution.

## 2 Materials and methods

### 2.1 Participants

This observational study was conducted at Hospital Universitari Sant Joan in Reus and included a subset of participants from the clinical trial registered at ClinicalTrials.gov (NCT05554224). The research included 87 participants systematically divided into three distinct groups. Cohort 1 served as the reference group and included 47 severely obese subjects with histologically confirmed normal liver architecture, defined by a NAS CRN score of 0, representing individuals with presumably healthy livers. Cohorts 2 and 3 consisted of the same 40 patients who were scheduled for sequential bariatric surgery due to insufficient weight loss or significant weight regain following their initial bariatric procedure. Cohort 2 represented these patients at baseline before their intervention, while Cohort 3 represented the same individuals after their first bariatric procedure, creating a paired comparison design that allowed for longitudinal assessment of treatment outcomes.

All participants were required to be 18 years of age or older. Exclusion criteria encompassed the presence of clinical or laboratory indicators of acute or chronic inflammation, active infectious diseases, and any cancer diagnosis.

The study protocol was designed and implemented in accordance with the ethical principles outlined in the Declaration of Helsinki. The investigation received approval from the Institutional Review Board under the references: PL4NASH(112/2021) and EOMPI2024(244/2024). All participants provided written informed consent prior to their inclusion in the study.

### 2.2 Sampling

Hepatic wedge biopsies were collected during bariatric surgery procedures and processed according to standardized institutional protocols. Tissue samples underwent formalin fixation followed by paraffin embedding. The paraffin blocks were sectioned at 4 μm thickness and prepared according to standard protocols for microscopic examination. Routine histological assessment was performed using Hematoxylin and Eosin (H&E) and Masson’s Trichrome staining, following automated, hospital-validated protocols.

Immune cell subpopulation analysis was performed by immunohistochemistry (IHC) using the fully automated Ventana BenchMark ULTRA platform (Roche Diagnostics, Indianapolis, IN, USA). A comprehensive antibody panel was applied to identify distinct immune cell populations, including Cluster of Differentiation 15 (CD15) for neutrophils, CD68 for macrophages, CD56 for natural killer cells, CD4 for CD4^+^ T lymphocytes, CD8 for CD8^+^ T lymphocytes, and CD163 for M2-polarized macrophages. Immunostaining was carried out using either the OptiView DAB IHC Detection Kit or the ultraView Universal DAB Detection Kit (Roche, Spain), according to antibody specific requirements. Signal detection was performed using DAB chromogen, followed by hematoxylin counterstaining. Quality control measures included the inclusion of both internal and external positive and negative controls in each staining batch. Detailed protocols are provided in **Table 1**.

**Table 1:** Summary of immunohistochemical staining protocols for each antibody using the Ventana BenchMark ULTRA system.

Antibody (cat. number)	Host species	Detection kit	Deparaffinization	Cell conditioning (antigen unmasking)	Antibody (primary)	Counterstain	Post-counterstain
CD15 (760–2504)	Mouse	OptiView DAB	Selected	ULTRA CC1, 124 min, 95 °C	16 min, 36 °C	Hematoxylin II, 16 min	Bluing, 8 min
CD68 (790–2931)	Mouse	ultraView DAB	Selected	ULTRA CC1, 20 min, 95 °C	20 min, 36 °C	Hematoxylin II, 16 min	Bluing, 8 min
CD56 (760-4596)	Rabbit	OptiView DAB	Selected	ULTRA CC1, 120 min, 100 °C	24 min, 36 °C	Hematoxylin II, 16 min	Bluing, 8 min
CD4 (790-4423)	Rabbit	OptiView DAB	Selected	ULTRA CC1, 80 min, 95 °C	16 min, 36 °C	Hematoxylin II, 16 min	Bluing, 8 min
CD8 (790-4460)	Rabbit	OptiView DAB	Selected	ULTRA CC1, 80 min, 95 °C	12 min, 36 °C*	Hematoxylin II, 16 min	Bluing, 8 min
CD163 (760-4437)	Mouse	ultraView DAB	Selected	ULTRA CC1, 172 min, 95 °C	32 min, 36 °C	Hematoxylin II, 16 min	Bluing, 4 min

*For CD8 protocol, after incubation with the primary antibody, the OptiView HQ Universal Linker was applied and incubated for 8 minutes, followed by the OptiView HRP Multimer for an additional 8 minutes.

### 2.3 Histological assessment

Histological assessment was performed by experienced pathologists, blinded to clinical data, according to NASH CRN criteria. Comprehensive liver biopsy analysis encompassed three key histopathological features that constitute the NASH CRN score. These included: 1) steatosis, graded from 0 to 3 based on the percentage of hepatocytes containing lipid vacuoles, 2) lobular inflammation, scored from 0 to 3 according to the number of inflammatory foci at 20x magnification and 3) hepatocellular ballooning, evaluated from 0 to 2 based on the presence and extent of hepatocyte hypertrophy and cytoplasmic alterations. Furthermore, hepatic fibrosis was staged according to Kleiner’s scoring system, with progressive gradation from F0 to F4.

MASH diagnosis was done following the composite NAS score, ranging from 0 to 8, and was calculated by summing these three key liver damage components (21).

### 2.4 Computational analysis of immunological cell populations

**Figure 1:**
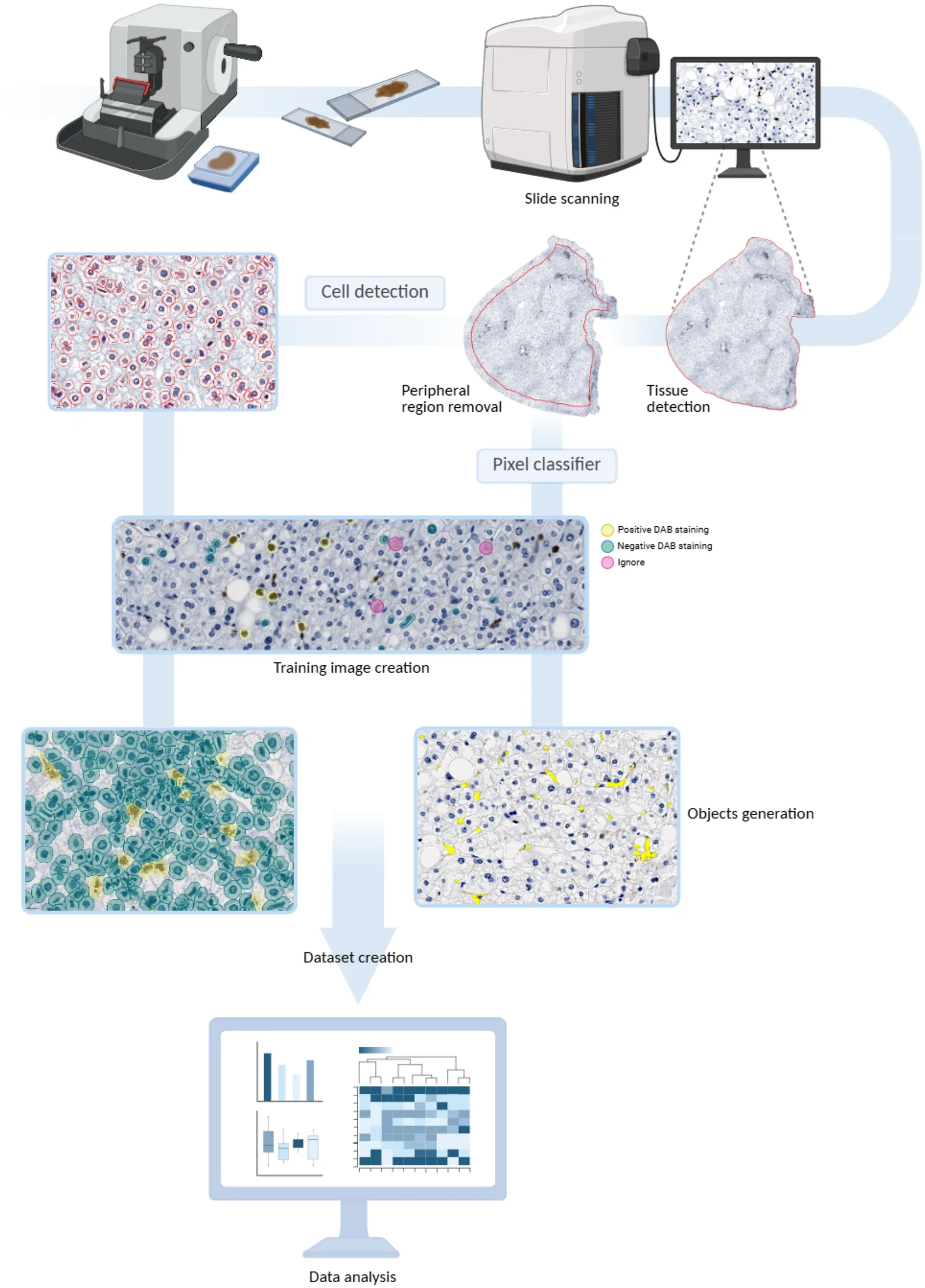
Schematic representation of QuPath methodology. Depending on the staining pattern two tools were used. For CD15 and CD8 the “Cell Detection” tool was applied and for CD68, CD4, CD163 and CD56 the “Pixel Classifier” tool was used. Created in BioRender.

As **Figure 1** depicts, a Ventana DP 200 device (Roche, Basel, Switzerland) was used for the slides digitalization at ×40 magnification. Image analysis was performed with QuPath software (Version 0.5.1). A project was created, and whole slide images were imported with the default H-DAB filter selected for immunohistochemical image analysis. To ensure accurate color representation, the “Estimate Stain Vectors” function was applied to each image, allowing for automatic color scale correction.

Tissue annotations were created manually to delineate whole tissue sections as regions of interest (ROI) and categorize them as tissue areas. To exclude non-representative or damaged peripheral regions, the “Expand Annotations” function was applied with an expansion radius of -100 µm. Artifacts within the tissue sections were manually corrected by erasing them or adjusting annotation regions as necessary.

Two primary analysis tools were employed based on the staining pattern observed. The “Cell Detection” tool was used for discrete cellular markers, which are characterized by well-defined cell boundaries with clear membrane and/or cytoplasmic localization. The “Pixel Classifier” tool was applied for diffuse or continuous staining patterns, characterized by overlapping marker distribution that does not conform to discrete cellular boundaries.

Individual cell identification and quantification in CD15 and CD8 immunostained sections were performed using the “Cell Detection” algorithm. The algorithm was configured with “Haematoxylin OD” detection parameters at a pixel resolution of 0.5 µm.

Default settings were maintained for all parameters, with the exception of the intensity threshold value, which was adjusted to 0.1. Following parameter optimization, cell objects were automatically generated. Subsequently, a comprehensive training macroimage was generated for each antibody, comprising 20 image sections. Each section contained 20 positive cells, 20 negative cells, and 20 artifacts designated for exclusion. These annotated training images were employed to develop and train antibody-specific “Object Classifiers”, which were then applied uniformly across all corresponding cohort images.

For antibodies exhibiting diffuse or continuous staining patterns (CD68, CD56, CD4, and CD163), training macro-images were created using identical annotation protocols (20 images with equivalent positive, negative, and artifact annotations). Individual “Pixel Classifiers” were developed for each antibody type; the Random Trees algorithm was consistently used as the machine learning classifier, while resolution parameters were optimized for each antibody-specific analysis, as detailed in **Table 2**. Other parameters remained as the default. Following pixel classification, tissue objects were systematically generated to enable downstream quantitative analysis.

**Table 2:** Pixel classifier resolution for each IHC stain.

IHC stain	Pixel classifier parameters - resolution
CD68	Full (0.25µm/pixel)
CD56	Very high (0.5µm/pixel)
CD4	High (1µm/pixel)
CD163	Very high (0.5µm/pixel)

Following application of either cell detection or pixel classification analysis, the created tissue objects were enhanced with spatial measurements using the “Analyze > Calculate Features > Add Shape Features” function to incorporate morphometric parameters into the quantitative analysis. Upon completion of the analysis, QuPath generated comprehensive quantitative datasets for each case. All image-associated data and analysis parameters were systematically saved and archived under respective patient identifiers to ensure data integrity and facilitate subsequent statistical analysis.

For CD15 and CD8 immunostained sections, positivity was quantified as the percentage of immunopositive cells relative to the total ROI area: (immunopositive cells area/total ROI area) × 100%. Positivity for diffuse staining patterns was expressed as a percentage calculated as: (DAB-stained area)/(total ROI area) × 100%.

### 2.5 Statistical analysis

All statistical analyses were conducted using R software (version 4.5.1) integrated with RStudio (version 2025.05.1.513). Since most variables did not follow a normal distribution, non-parametric statistical methods were utilized throughout the analysis.

For comparisons of continuous variables between Cohort 1 and the other two cohorts, the Wilcoxon rank-sum test was used. Associations between categorical variables were assessed using Chi-square test. For paired comparisons between Cohort 2 and Cohort 3, the Wilcoxon signed-rank test was applied to continuous variables, and McNemar’s test was used for categorical variables. Correlations between continuous variables were assessed using Spearman’s rank correlation coefficient. A statistical significance threshold was set at *p* ≤ 0.05 for all analyses. Descriptive statistics for continuous data were presented as medians with interquartile ranges (IQRs), while categorical data were expressed as frequencies and percentages. Data visualizations and graphical outputs were created using R and further enhanced with Inkscape software (version 1.4.2) to improve figure quality and presentation.

## 3 Results

### 3.1 Selected participants

The study included 87 participants across three cohorts (**Table 3**): 47 severely obese patients without liver alterations serving as controls (Cohort 1), 40 severely obese patients with significant baseline liver pathology (Cohort 2), and the same 40 patients from Cohort 2 following the first bariatric surgery (Cohort 3).

**Table 3:** Selected characteristics of the study population.

	Cohort 1 n = 47	Cohort 2 n = 40	Cohort 3 n = 40
Women, n (%)	34 (72.3)	32 (80.0)	32 (80.0)
Age, years	45 (40-58)	43 (39-49)	49 (41-54)^c^
BMI, kg/m^2^	42.4 (39.2-46.3)	53.1 (47.7-57.7)^a^	40.6 (37.0-43.0)^b,c^
T2DM, n (%)	9 (19.1)	12 (30.0)	2 (5.0)^c^
Hypertension, n (%)	19 (40.4)	17 (42.5)	9 (22.5)^c^
Dyslipidemia, n (%)	6 (12.8)	11 (27.5)	6 (15.0)
Hepatic histologic features			
Steatosis score, n (%)			
<5%	47 (100.0)	4 (10.0)	31 (77.5)
5 – 33%	0	19 (47.5)	9 (22.5)
34 – 66%	0	12 (30.0)	0
>66%	0	5 (12.5)^a^	0^b,c^
Hepatocellular ballooning, n (%)			
None	47 (100.0)	1 (2.5)	19 (47.5)
Few cells	0	9 (22.0)	12 (30.0)
Many cells	0	30 (75.0)^a^	9 (22.5)^b,c^
Lobular inflammation, n (%)			
No foci	47 (100.0)	0	0
<2 foci	0	1 (2.5)	2 (5.0)
2–4 foci	0	21 (52.5)	19 (47.5)
>4 foci	0	18 (45.0)^a^	19 (47.5)^b^
Fibrosis, n (%)			
None	19 (40.4)	0	0
Perisinusoidal or periportal fibrosis	19 (40.4)	0	4 (10.0)
Perisinusoidal and periportal fibrosis	7 (14.9)	19 (47.5)	24 (60.0)
Bridging fibrosis	2 (4.3)	20 (50.0)	11 (27.5)
Cirrhosis	0	1 (2.5)^a^	1 (2.5)^b,c^
MASH diagnosis			
Non-MASH	47 (100.0)	0	9 (22.5)
Uncertain	0	2 (5.0)	25 (62.5)
MASH	0	38 (95.0)^a^	6 (15.0)^b,c^

BMI, Body mass index; T2DM, Type 2 diabetes mellitus. Significant differences (p ≤ 0.05 or lower) in comparisons are indicated by: ^a^ Cohort 1 vs. Cohort 2. ^b^ Cohort 1 vs. Cohort 3 and ^c^ Cohort 2 vs. Cohort 3.

Women comprised the majority of participants across all cohorts, ranging from 72.3% in Cohort 1 to 80.0% in Cohorts 2 and 3. Median age was comparable between baseline cohorts 1 and 2, with a median interval of 6 years between the pre- and post-surgical assessments. BMI differed significantly between groups, with Cohort 2 demonstrating the highest BMI at 53.1 kg/m² compared to 42.4 kg/m² in Cohort 1 and 40.6 kg/m² in Cohort 3, reflecting statistically significant weight reduction following bariatric intervention.

Comorbidity profiles varied substantially among cohorts. Cohort 2 showed a higher prevalence of dyslipidemia and type 2 diabetes mellitus compared to Cohort 1, with statistically significant improvement in T2DM and hypertension following bariatric surgery.

Hepatic histological features revealed the expected spectrum from normal to pathological states across cohorts. Cohort 1 exhibited no steatosis, hepatocellular ballooning, or lobular inflammation by design. Conversely, Cohort 2 demonstrated severe hepatic alterations, with 12.5% of patients exhibiting steatosis exceeding 66%, 75.0% showing hepatocellular ballooning in many cells, and 45.0% presenting more than 4 inflammatory foci per field. Post-surgical patients in Cohort 3 demonstrated marked histological improvement, with 77.5% achieving steatosis below 5% and substantial reductions in hepatocellular ballooning and lobular inflammation. Fibrosis patterns reflected disease progression and post-surgical resolution across the study cohorts, with significant worsening from Cohort 1 to Cohort 2 and marked improvement between pre and post-intervention assessments. Regarding MASH diagnosis, 95.0% of Cohort 2 patients met diagnostic criteria compared to only 15.0% in the post-surgical cohort, demonstrating significant improvement following bariatric intervention.

### 3.2 Hepatic immune cell infiltration and composition following bariatric surgery

**Figure 2:**
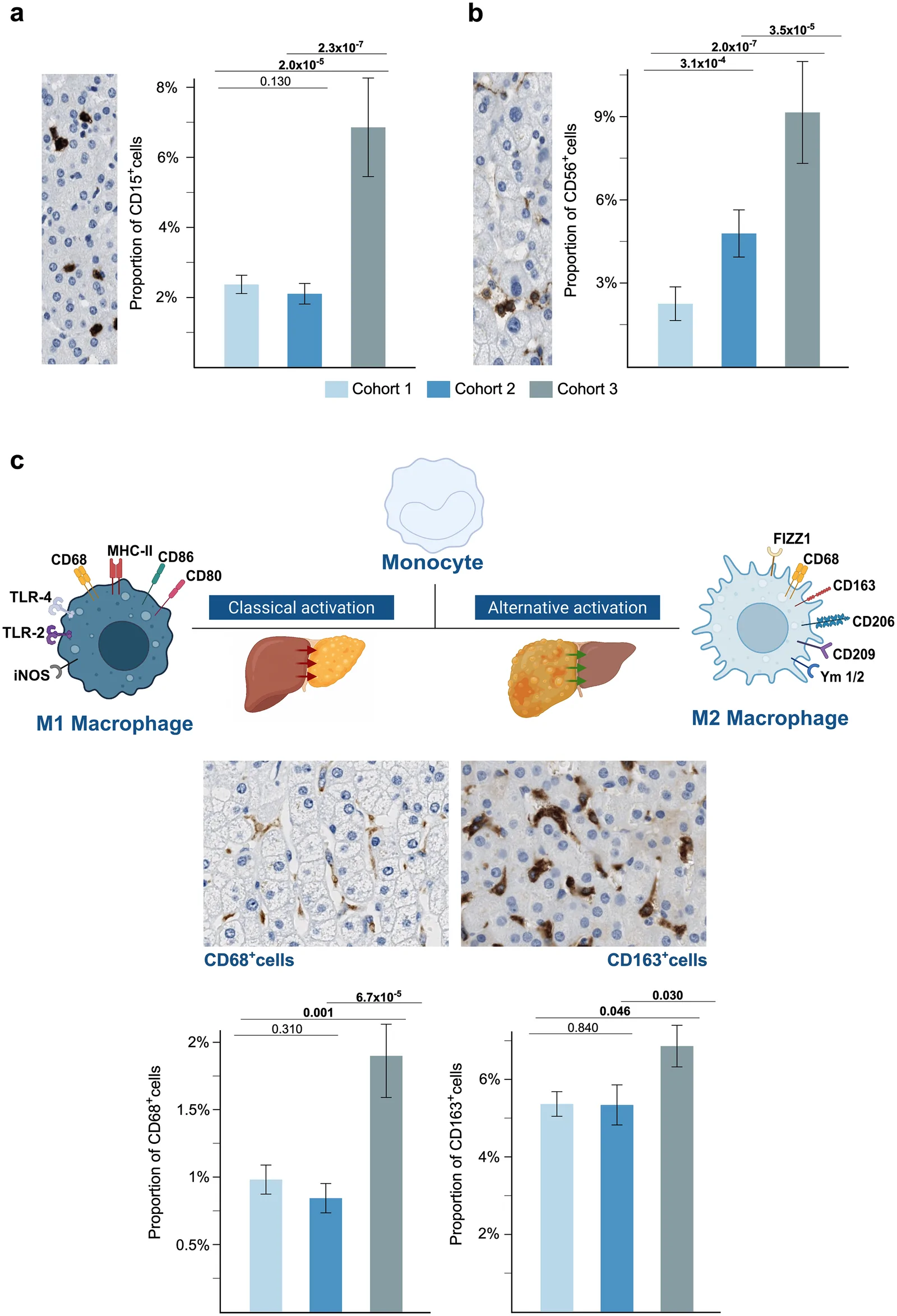
Characterization of the innate immune system across patient cohorts. **(a)** Representative histological image (10x magnification) of CD15^+^ cells and corresponding bar plot showing a statistically significant increase in their abundance after bariatric surgery. **(b)** Representative 10x histological section of CD56^+^ cells and bar plot illustrating their progressive increase across cohorts. Created in BioRender. **(c)** Schematic representation of macrophage polarization from monocytes toward the M1 and M2 phenotypes, including key marker expression. Representative histological images (10x magnification) of CD68^+^ and CD163^+^ cells and corresponding bar plots showing differences in their abundance across cohorts.

Immunohistochemical analysis revealed distinct CD15^+^, mainly neutrophils, infiltration patterns across patient cohorts (**Figure 2a**). Severely obese patients without hepatic damage (Cohort 1) exhibited baseline CD15^+^ cell proportions of approximately 2.4% of the total tissue, which remained similar in pre-surgical patients presenting liver damage (Cohort 2) with a 2.1% proportion. Long-term post-surgical evaluation (Cohort 3) demonstrated a statistically significant increase to 6.8% compared to both baseline groups with a *p*-value < 0.001, indicating sustained neutrophilic inflammation at a median 6 years post-sleeve gastrectomy.

**Figure 2b** depicts the CD56^+^ cell analysis which revealed progressive accumulation patterns across patient cohorts. Severely obese patients without hepatic alterations demonstrated minimal CD56^+^ natural killer cell presence at approximately 2.2% of total tissue. Pre-surgical liver injury was associated with a significant increase in CD56^+^ cell presence to approximately 4.8% (*p*-value = 3.1×10^−4^ versus Cohort 1), indicating acute natural killer cell recruitment in liver damage progression. Long-term post-surgical evaluation showed the most dramatic CD56^+^ cell accumulation, reaching a 9.1% of total tissue (*p*-value = 2.0×10^−7^ versus Cohort 2; *p*-value = 3.5×10^−5^ versus Cohort 1). This increase from baseline suggests sustained natural killer cell activation and recruitment that intensifies over the post-surgical period, potentially reflecting ongoing immune surveillance and tissue remodeling processes.

Panel c illustrates the macrophage polarization spectrum representing a functional continuum where circulating monocytes differentiate into distinct phenotypic states upon tissue infiltration. Classical activation generates M1 macrophages characterized by proinflammatory properties, enhanced pathogen recognition, and production of inflammatory cytokines that promote tissue damage and antimicrobial responses. Alternative activation produces M2 macrophages exhibiting anti-inflammatory and tissue-remodeling functions, promoting wound healing, angiogenesis, and extracellular matrix remodeling (25, 26).

Immunohistochemical analysis using CD68 as a general macrophage marker and CD163 for M2-specific identification revealed distinct polarization patterns across patient cohorts. CD68^+^ cells showed minimal presence in severely obese patients without hepatic damage (1.0%) and remained stable in severely obese patients with liver injury (0.8%), but significantly increased in long-term post-surgical patients compared to those with a NASH CRN of 0 reaching a 1.9% of the total tissue (*p*-value = 0.001 versus Cohort 1) and with themselves at baseline (*p*-value = 6.7x10–5 versus Cohort 2). CD163^+^ M2-like macrophages demonstrated progressive accumulation across cohorts, with Cohort 1 showing 5.4% of total cells, which remained stable in Cohort 2. Long-term follow-up (Cohort 3) revealed increased M2 macrophage infiltration to 6.7% of total cells (*p*-value = 0.046 versus Cohort 1; *p*-value = 0.030 versus Cohort 2), indicating enhanced tissue remodeling responses during chronic post-surgical adaptation.

**Figure 3:**
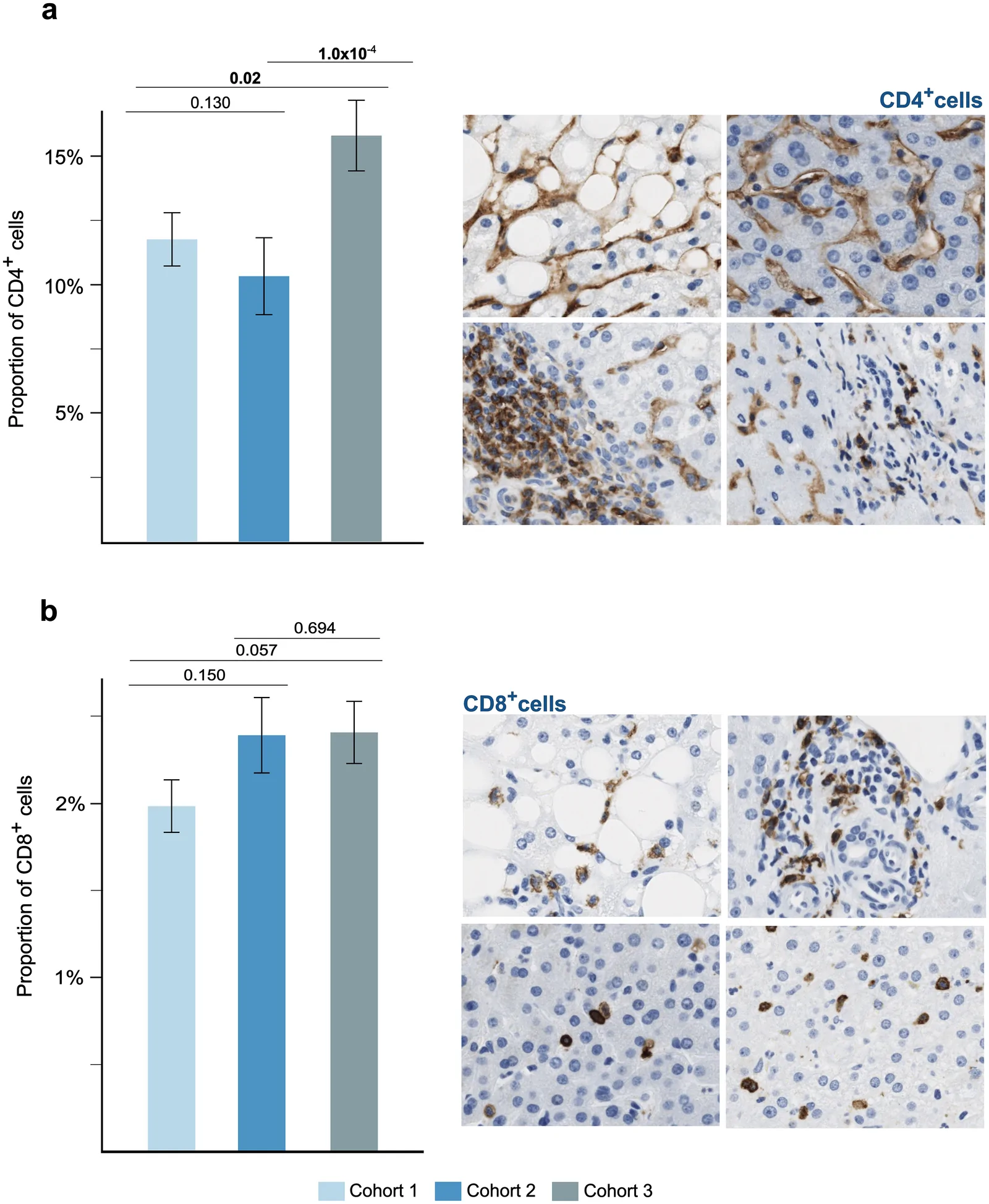
Adaptive immune response in liver damage and post-bariatric surgery. **(a)** Bar plot showing the differences in CD4^+^ cell abundance, accompanied by representative histological images (10x magnification) illustrating diffuse CD4 immunostaining. **(b)** Bar plot showing the absence of significant differences in CD8^+^ cell abundance between cohorts, with representative CD8 IHC images at 10x magnification.

T-lymphocyte infiltration patterns varied significantly across patient cohorts (**Figure 3**). CD4^+^ cells demonstrated a significant increase following sleeve gastrectomy, reaching 15.8% of total tissue (Cohort 3) compared to baseline levels in Cohort 1 (11.8%) and Cohort 2 (10.3%). Immunohistochemical staining revealed a diffuse distribution pattern with CD4^+^ cells present both as discrete lymphocytes and within hepatic sinusoids (**Figure 3a** right). This substantial elevation represents sustained CD4^+^ T lymphocytes recruitment during long-term post-surgical adaptation.

CD8^+^ T lymphocyte infiltration demonstrated stable levels across patient cohorts with no statistically significant differences (**Figure 3b**). Baseline severely obese patients without hepatic alterations (Cohort 1) exhibited approximately 2.0% of total tissue comprising CD8^+^ cells, which showed minimal variation in pre-surgical patients with liver injury (Cohort 2, 2.4% of total tissue) and remained consistent in long-term post-bariatric surgery patients (Cohort 3, 2.4% of total tissue).

To explore the clinical relevance of these findings, immune-cell proportions were analyzedaccording to the presence of type 2 diabetes mellitus, hypertension, and dyslipidemia (**Supplementary Figure 1**). No significant differences were observed, except for a lower proportion of CD68^+^ cells in patients with dyslipidemia (*p* = 0.03). Changes in immune-cell populations showed generally weak correlations with BMI reduction, with only the change in CD56^+^ cells reaching statistical significance. Changes in CD15^+^ cells showed modest correlations with changes in the inflammation score (ρ = −0.46, *p* = 0.003) and the NASH CRN score (ρ = −0.33, *p* = 0.043). These analyses were considered exploratory.

### 3.3 Immune microenvironment alterations and immunohistochemical predictive performance for liver damage

**Figure 4:**
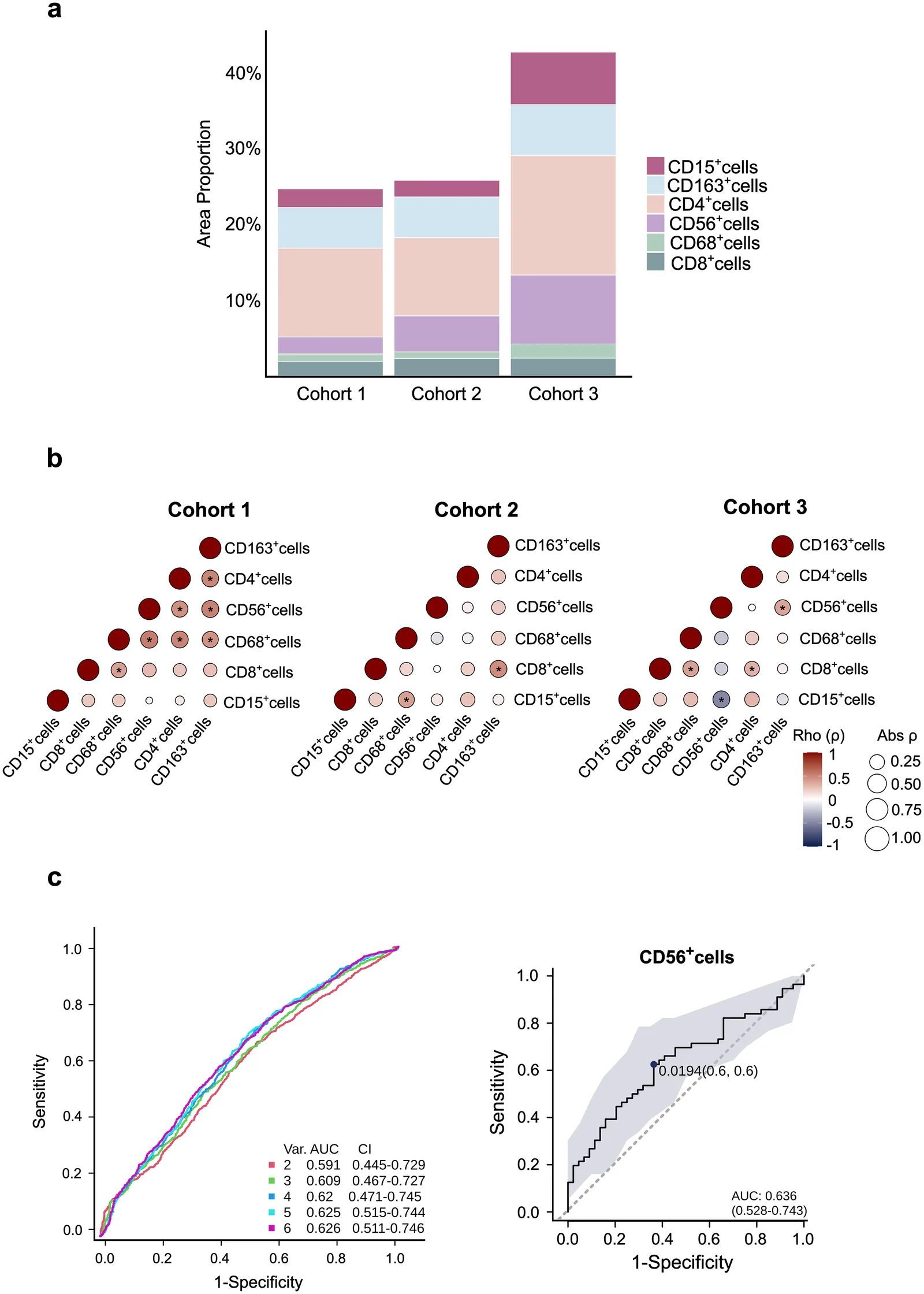
Immune system reorganization across clinical conditions. **(a)** Bar plot illustrating the abundance and distribution patterns of the different immune markers across cohorts. **(b)** Correlation plots showing the relationships between immune markers within each cohort (* indicating a p-value < 0.05). **(c)** Immune markers show no ability to distinguish between MASH and non-MASH patients.

As illustrated in **Figure 4a**, the observed changes extend beyond proportional differences in tissue representation, reflecting fundamental alterations in immune cell composition across patient cohorts. Comprehensive analysis of immune cell distribution patterns revealed distinct hierarchical arrangements that varied markedly between clinical scenarios.

In severely obese patients without liver injury (Cohort 1), the distribution of immune cells followed a clear ascending hierarchy: CD68^+^ < CD8^+^ < CD56^+^ < CD15^+^ < CD163^+^ < CD4^+^. In contrast, pre-surgical patients with liver damage (Cohort 2) showed a different distribution: CD68^+^ < CD15^+^ < CD8^+^ < CD56^+^ < CD163^+^ < CD4^+^.

Notably, long-term post-bariatric surgery patients (Cohort 3) displayed a distinct immunological profile: CD68^+^ < CD8^+^ < CD163^+^ < CD15^+^ < CD56^+^ < CD4^+^. This cohort also exhibited the highest overall abundance of positive cells, with statistically significant differences when compared to both Cohort 1 and Cohort 2 (*p*-value < 0.001). These variations in immune cell composition suggest that the hepatic immune microenvironment undergoes substantial reconfiguration across the three groups, with Cohort 3 exhibiting unique immune characteristics, despite the persistence of severe obesity after surgery and concurrent improvements in liver histology.

Beyond individual cell population changes, inter-cellular correlations between different immune cell types varied significantly among the three cohorts, providing evidence for distinct immunological processes and cellular cross-talk patterns operating within each clinical context (**Figure 4b**). These correlation matrices suggest that immune cell recruitment and activation may occur through different pathways depending on disease stage and surgical intervention status, potentially reflecting varying degrees of inflammatory resolution and tissue remodeling processes.

Despite these marked qualitative and quantitative differences in immune marker distribution patterns, the ability of these immunohistochemical parameters to discriminate between hepatic states proved limited. Multivariate analysis incorporating all immune markers yielded a maximum area under the curve (AUC) of 0.626 (**Figure 4c**), indicating modest discriminative capacity. Similarly, evaluation of the single most discriminative immunohistochemical marker, CD56 achieved an AUC of 0.636 (the right-hand ROC plot in **Figure 4d**); with both values indicating limited separation between hepatic states. These findings suggest that while immune cell composition undergoes substantial reorganization across disease stages, individual immune markers lack sufficient specificity and sensitivity for reliable MASH diagnosis in this patient population.

## 4 Discussion

This study provides comprehensive evidence that the hepatic immune microenvironment undergoes stage-specific alterations throughout the progression of obesity-related liver disease and following recovery after bariatric intervention. The documented changes extend beyond simple quantitative shifts in immune cell populations to encompass fundamental reorganization of cellular hierarchies and inter-cellular network dynamics, revealing previously unrecognized complexity in hepatic immunological responses to bariatric surgery.

Consistent with established literature, the vast majority of patients showed substantial improvements in liver architecture following bariatric intervention (27, 28), even though they remained in a state of severe obesity. However, contrary to expectations that immune activity would decline in parallel with histological recovery (29, 30), our findings revealed a paradoxical persistence of immune cell infiltration that remained elevated during tissue healing and at comparable levels in severely obese patients irrespective of liver damage status, challenging the traditional association between tissue injury and immune activation.

Specifically, the elevation of neutrophils and natural killer cells postoperatively suggested that hepatic repair involves persistent inflammatory mechanisms rather than simple immune quiescence (31). While the presence of CD15^+^ cells remained stable with disease severity from Cohort 1 to Cohort 2, the almost threefold increase seen in Cohort 3 may initially seem counterintuitive, particularly given the typically pro-inflammatory role of neutrophils in liver pathology through mechanisms such as neutrophil extracellular traps (NETs), elastase, myeloperoxidase, proteinase 3 release, and the generation of reactive oxygen species (ROS) (32, 33). However, this sustained neutrophil presence may reflect a functional reprogramming toward tissue repair and homeostasis maintenance. This may occur through the release of regulatory microRNAs, such as miRNA-223, which encourages M2 macrophage polarization and fosters an anti-inflammatory tissue environment (34–37). Additionally, neutrophils may contribute to extracellular matrix remodeling through their secretion of metalloproteinases, which could contribute to the improvement of fibrosis (38).

Similarly, the progressive accumulation of CD56^+^ NK cells across all three cohorts reflects a dynamic immune response evolving with disease progression and recovery. The increase from Cohort 1 to Cohort 2 could represent an adaptive response to hepatocellular stress, where NK cells exhibit a dual inflammatory role: amplifying hepatic inflammation through robust secretion of proinflammatory cytokines and eliminating lipotoxic-damaged hepatocytes and activated stellate cells (HSCs) through cytotoxic mechanisms (39–42). Following bariatric intervention, sustained NK cell elevation in Cohort 3 suggests functional reprogramming toward tissue restoration, with cells adopting regulatory phenotypes that promote fibrosis resolution and hepatocyte regeneration through targeted cytokine secretion, such as platelet-derived chemokine 7 (CXCL7) and IL-22 (43, 44). However, without phenotypic characterization and spatial validation we cannot definitively exclude the possibility of continued pro-inflammatory NK activity.

The macrophage compartment analysis reveals sophisticated immune adaptation mechanisms. The comparable CD68^+^ and CD163^+^ (M2 macrophages (25, 45) presence across Cohorts 1 and 2, combined with the statistically significant increase after bariatric intervention, suggests preferential polarization toward alternatively activated macrophages involved in tissue repair and remodeling rather than classical inflammatory responses. This M2-dominant pattern in post-surgical patients supports the concept that successful bariatric intervention shifts hepatic immunity toward resolution and repair rather than perpetuating inflammatory damage (18, 26, 46).

Analysis of adaptive immunity revealed a selective pattern of immune modulation: while CD8^+^ cytotoxic T cell populations remained stable across disease stages and following surgical intervention, CD4^+^ T cells showed substantial post-surgical accumulation. This differential response suggests selective activation of immune coordinating functions rather than cytotoxic effector mechanisms. However, given the heterogeneity of CD4^+^ T cell subsets, such as regulatory T cells (Tregs), T helper 1 (Th1), and T helper 17 (Th17) cells, each with context-dependent beneficial or detrimental roles, the observed increase cannot definitively indicate whether this response is protective or pathogenic without further subset characterization (47–50).

The persistent immune cell infiltration observed in post-bariatric patients, together with the reorganization of immune cell hierarchies and correlations, represents a novel and clinically relevant finding. Rather than reverting to a preoperative or baseline immune profile, successful bariatric intervention appears to induce a distinct immunological “third state.” This state is qualitatively different from both healthy obese and pathological conditions. As a hypothesis-generating investigation, our study establishes the descriptive foundation for identifying and characterizing this distinct immune configuration, but determining whether this state represents an optimized, neutral, or potentially maladaptive immune response requires further mechanistic investigation.

The limited associations with BMI reduction may partly reflect that most patients remained severely obese despite substantial postoperative weight loss. Moreover, the absence of consistent differences according to metabolic comorbidities suggests, without establishing causality, that the observed immune remodeling may be more closely related to the surgical intervention and/or hepatic recovery than to the comorbidity profile itself.

The modest discriminatory performance of immunohistochemical markers reveals an important disconnect between immunological mechanisms and conventional diagnostic frameworks. This limited discriminatory capacity may not represent a shortcoming of immune profiling per se but rather highlights fundamental limitations of traditional NAS CRN-based definitions of MASH. The NAS CRN scoring system relies on visual, semi-quantitative assessment with inherent intra and inter-observer variability, whereas our immunohistochemical analysis quantifies continuous variables representing actual immune cell densities (51, 52). This methodological incongruence, comparing categorical histological scores against continuous immune cell measurements, explains why substantial immune remodeling occurs independently of traditional histological classifications. Given that our findings demonstrate that immune dynamics are decoupled from classical injury patterns, current diagnostic criteria established without consideration of immune components may be insufficiently nuanced to capture the full spectrum of immune-mediated metabolic liver disease.

Several limitations warrant consideration. The cross-sectional design prevents definitive establishment of causal relationships between immune changes and clinical outcomes. The median 6-year interval between pre- and post-surgical assessments, while providing valuable long-term insights, introduces potential confounding from temporal factors independent of surgical intervention. Additionally, the focus on severely obese populations may limit generalizability to broader patient populations with less extreme metabolic dysfunction.

These findings fundamentally expand understanding of hepatic immune responses in obesity-related liver disease and post-bariatric surgery adaptation. The demonstration of persistent immune activation years post-surgically, despite metabolic and histological improvement, suggests that bariatric surgery creates a unique immunological state requiring long-term monitoring and potentially targeted therapeutic approaches. Future research should focus on determining whether persistent immune activation represents beneficial adaptation or poses risks for long-term hepatic health, and whether therapeutic modulation of post-surgical immune responses could optimize patient outcomes.

The complex interplay between metabolic improvement, histological resolution, and sustained immune activation revealed in this study underscores the need for comprehensive, longitudinal immune profiling in post-bariatric surgery patients to fully understand the long-term consequences of surgical metabolic interventions on hepatic immunity and the underlying mechanisms driving tissue recovery and metabolic adaptation.

## Data Availability

The datasets generated and/or analyzed during the current study are available from the corresponding author upon reasonable request.

## Supplementary material

Supplementary Figure 1 Clinical and histological associations of hepatic immune-cell changes. **(a)** Hepatic immune-cell proportions according to type 2 diabetes mellitus, hypertension, and dyslipidemia. **(b)** Spearman correlations between ΔBMI (1^st^ intervention BMI – 2^nd^ intervention BMI) and paired changes in immune-cell proportions (2^nd^ intervention cell proportion – 1^st^ intervention cell proportion). **(c)** Correlations between changes in CD15^+^ cells and changes in inflammation score and NASH CRN/NAS scores. Values indicate Spearman’s ρ; * indicating a p-value < 0.05.
